# Imaging insights of FDG-PET from neonates to infants

**DOI:** 10.1007/s11604-025-01763-z

**Published:** 2025-03-05

**Authors:** Ryogo Minamimoto, Yumi Abe, Shinichiro Kamiya, Toshiki Nakane, Rintaro Ito, Katsuhiko Kato, Shinji Naganawa

**Affiliations:** 1 Department of Integrated Image Information Analysis, Nagoya University Graduate School of Medicine, 65 Tsurumaicho, Shouwa-ku, Nagoya, Aichi 466-8550 Japan; 2 Department of Radiology, Nagoya University Graduate School of Medicine, Nagoya, Aichi Japan; 3 Department of Innovative Biomedical Visualization (Ibmv), Nagoya University Graduate School of Medicine, Nagoya, Aichi Japan; 4 Functional Medical Imaging, Biomedical Imaging Sciences, Division of Advanced Information Health Sciences, Department of Integrated Health Sciences, Nagoya, Aichi Japan

**Keywords:** FDG, PET, PET/CT, Neonates, Infants, Physiological variations, Brain

## Abstract

In pediatric oncology, ^18^F-fluorodeoxyglucose (FDG)-positron emission tomography (PET)/computed tomography (CT) is valuable as a tool for noninvasive imaging and monitoring. While many reports have reviewed the use of PET and PET/CT in pediatrics, considerable variations in age, body size, and metabolism are seen during different stages of childhood development. Neonates (from birth to one month old) and infants (from 1 month to 1 year) present unique challenges for FDG-PET/CT examination due to their small body size, the immaturity of organs, the need for specialized patient preparation, and support requirements during scanning. In addition, differences in metabolic activity can lead to distinct differences in patterns of physiological FDG uptake on PET/CT imaging between neonates and infants. These factors differ significantly from those encountered in older children, who may be treated similarly to adults during imaging procedures. This review, based on both the literature and clinical experience, explores the specific characteristics, challenges, and considerations for FDG-PET/CT imaging from neonates to infants, with a focus on optimizing imaging protocols and interpreting physiological variations in this growth period.

## Introduction

In children, ^18^F-fluorodeoxyglucose (FDG)-positron emission tomography (PET)/computed tomography (CT) has become a critical tool for the less-invasive evaluation and management of malignant diseases in children. These imaging modalities provide valuable insights into metabolic activity, allowing for precise disease staging, treatment monitoring, and detection of recurrence. While the use of FDG-PET/CT in pediatric oncology has been widely studied and reviewed, the unique challenges present in imaging neonates and infants have received less attention.

Neonates and infants are in a critical phase of rapid growth and development, with significant physiological differences compared to older children and adults. These differences include variations in organ maturity, metabolic rates, and body composition, all of which can affect FDG distribution and uptake patterns. Moreover, the practical aspects of imaging in this growth period, such as patient preparation, maintaining stillness during scanning, and the handling of small body sizes, introduce additional complexities. Understanding these factors is essential for optimizing FDG-PET/CT protocols and ensuring accurate image interpretation in neonates and infants.

This review aims to explore the specific characteristics of FDG-PET/CT imaging in neonates and infants, highlighting the physiological and technical considerations that distinguish this population from older children and adults. Since FDG-PET/CT examinations were performed to monitor underlying conditions, the images in this review may have been influenced by the patients’ background diseases and treatments. Nonetheless, we have highlighted the common characteristics of FDG-PET/CT imaging in neonates and infants, focusing on shared patterns within these cases and supporting their validity with foundational knowledge.

## Patient preparation

Marked variability in blood glucose levels and transient alterations in the insulin secretion set point are common during the first few hours of life. However, by approximately 72 h of age, healthy term neonates typically achieve fasting blood glucose levels comparable to those seen in children and adults. Normal fasting blood glucose levels in neonates and infants are maintained within a narrow range (approximately 60–100 mg/dl) despite frequent feeding and fasting cycles [1]

For an FDG-PET/CT study, subjects are generally required to fast for 4–6 h before the scan. In addition, subjects are recommended to drink water to maintain hydration, especially if anesthesia or sedation is not involved [2–4]. For infants, the EANM guideline stated that injection of the radiotracer should be timed as close to the next breast/milk feeding, with feeding may resume as early as 30 min after the injection [3, 4]. One study evaluated the effects of preoperative fasting on plasma glucose levels and gastric emptying in infants less than 3 months old after feeding with either breast milk or infant formula [5]. The results showed that prior to anesthesia induction, no infants were hypoglycemic, where hypoglycemia was defined as a plasma glucose level of less than 40 ml/dl. However, 5% of infants had a significant volume of residual gastric contents. Mean intraoperative plasma glucose levels rose significantly in response to the stress of anesthesia and surgery. Infants in this age group generally easily tolerate a 3- to 4-h preoperative fast, as no cases of intraoperative hypoglycemia were observed [5]. These findings suggest that routine intraoperative glucose supplementation may not be necessary, although plasma glucose should still be monitored. For surgery, various anesthesia societies recommend minimal fasting times for gastric emptying: 1–2 h for clear liquids, 3–4 h for breast milk, and 4–6 h for infant formula and nonhuman milk [6]. Based on this knowledge, fasting protocols for FDG-PET in neonates and infants should be individualized according to the patient’s condition and the judgment of the attending specialists.

While the “SNMMI/EANM guideline for pediatric FDG-PET/CT for Oncology” does not recommend the routine use of anesthesia during PET studies, institutional policies should be followed if sedation or general anesthesia is deemed necessary. General anesthesia must be administered after tracer injection and before image acquisition [5, 7, 8]. However, anesthesia can effect on regional cerebral glucose metabolism [8, 9]. Significant glucose hypometabolism was detected bilaterally in the medial parieto-occipital cortex, encompassing the lingual gyrus, cuneus, posterior cingulate, and middle occipital gyrus in sedated children [9].

The risks of sedation include hypoventilation, apnea, airway obstruction, cardiopulmonary arrest, and the morbidity and mortality associated with these complications. The decision to sedate a child must involve careful consideration of the potential risks and benefits, with proper preparation to minimize the likelihood of such adverse events.

In nuclear medicine, sedation offers the advantage of reducing patient motions during prolonged imaging acquisitions, facilitating the successful completion of procedures that require the cooperation of older children who may find it difficult to comply [10]. In addition, sedation enhances patient care by minimizing discomfort. Judicious use of sedation is therefore crucial to maximize the quality of imaging procedures while minimizing associated risks. Comfortable immobilization devices, such as vacuum mattresses, cushions, sandbags, safety straps, and immobilization splints or pads, may be helpful for achieving stable immobilization and positioning of neonates and infants, enabling smooth PET image acquisition without motion artifacts [8, 11].

**Fig. 1:**
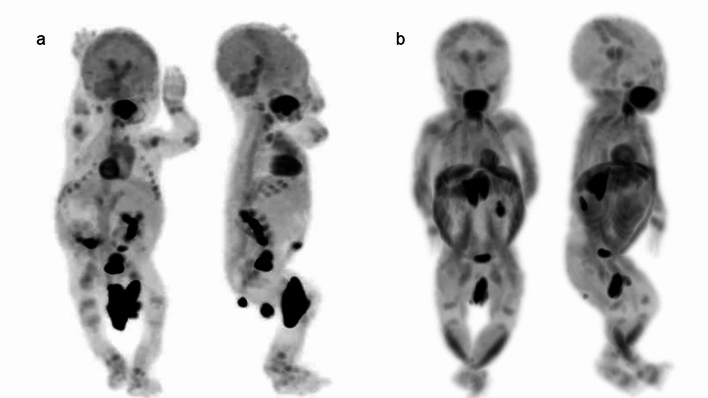
Whole body FDG-PET images in neonates, **a** 9 days after birth, **b** 15 days after birth. FDG uptake is concentrated in the sensorimotor cortex, thalamus, brainstem, cerebellar vermis and hemispheres (**a**, **b**). Increased uptake in tongue muscles is due to pacifier sucking (**a**, **b**). Physiological uptake is seen in the thymus (**a**). Abdominal muscle and diaphragm uptake reflect breathing support. Uptake in neck, upper, and lower extremity muscles results from uncontrolled movements around FDG injection (**b**). Various FDG uptake patterns in the bladder and FDG-containing urine in diapers are observed in neonates (**a**, **b**). Band-like FDG uptake is observed in the growth plates (**a**, **b**)

**Fig. 2:**
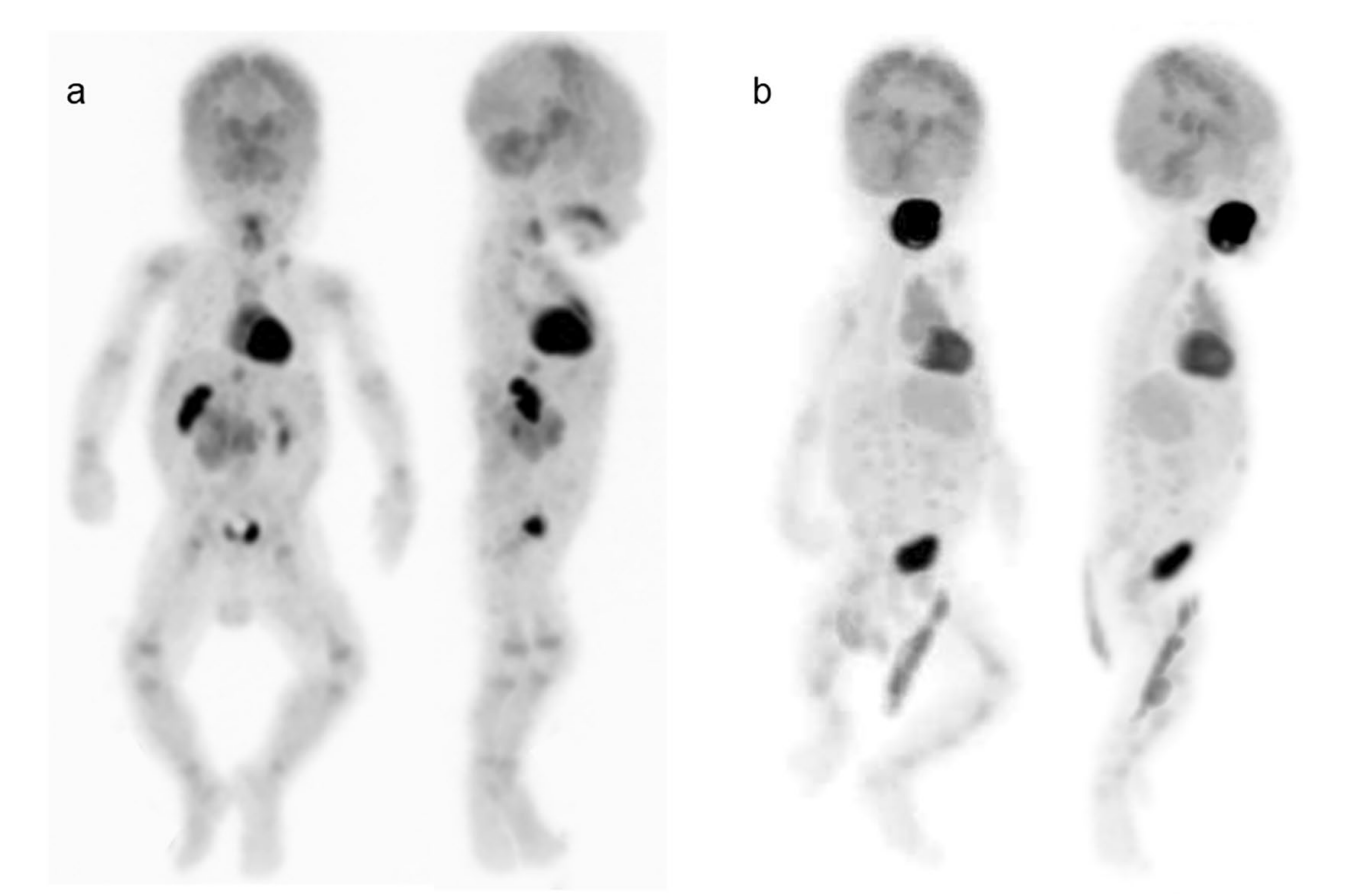
Whole body FDG-PET images in infants, **a** 31 days after birth, **b** 38 days after birth. FDG uptake in infants’ brains resembles that of neonates, with a slight increase in brain hemisphere (**a**, **b**). The other features are the same as those in neonates, as shown in Fig. 1

**Fig. 3:**
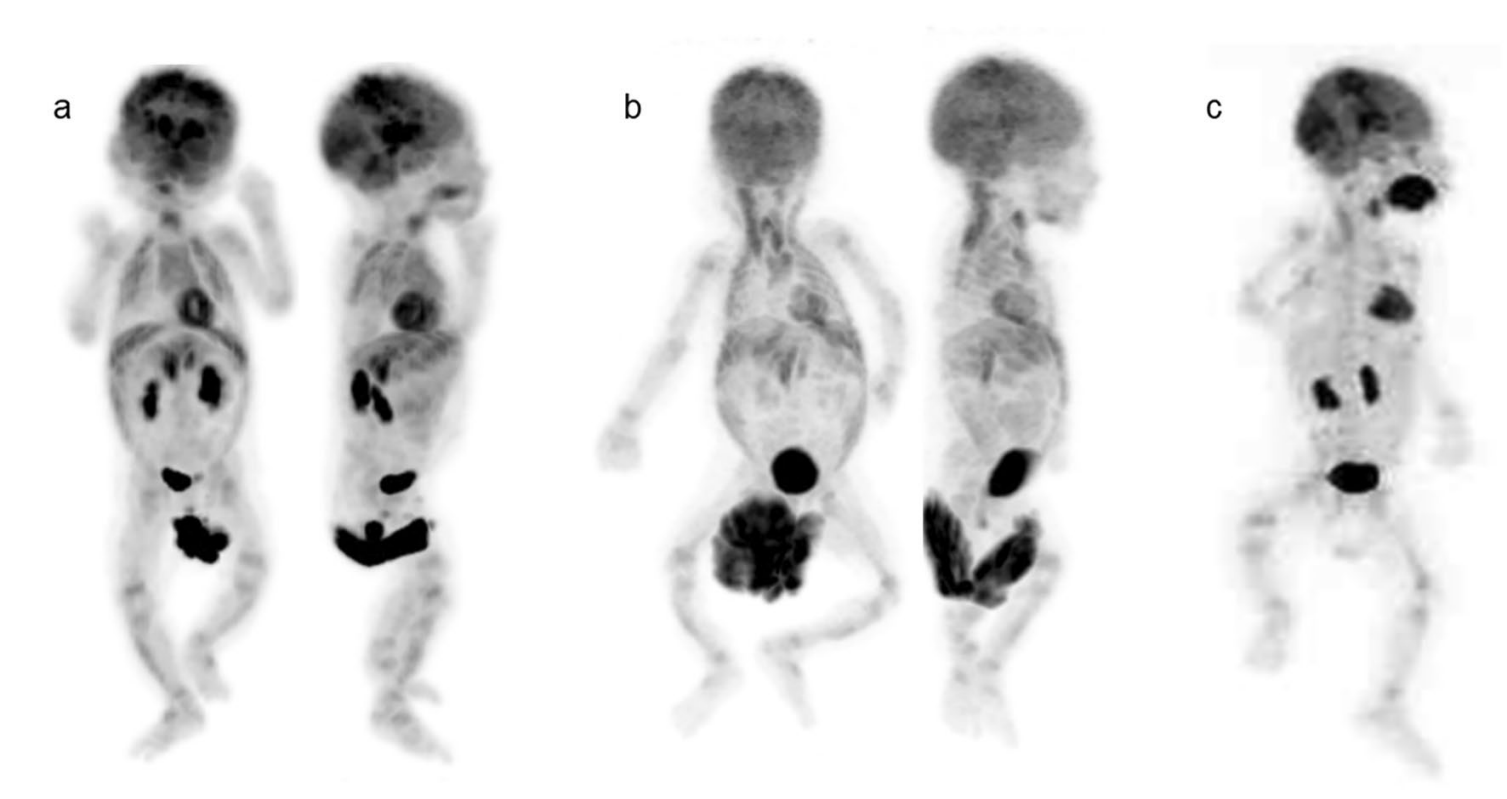
Whole body FDG-PET images in infants, **a** 61 days after birth, **b** 83 days after birth, **c** 107 days after birth**.** By 2 months after birth, FDG uptake in the brain hemispheres becomes more prominent, approaching adult brain levels (**a**–**c**). In addition to the abdominal muscles and diaphragm, FDG uptake in the intercostal muscles begins to appear, reflecting breathing support (**a**, **b**)

However, advances in PET technology such as digital PET/CT and total body PET/CT have allowed for much shorter imaging times with lower injection doses [12, 13], so the need for sedation during PET may be less critical than in the past. In FDG-PET, the distribution of the tracer can be influenced by various behaviors before or after injection. FDG uptake into muscle tissue can occur under several clinical conditions, so children should avoid exercising, talking, or chewing after injection, but such voluntary control is not feasible in neonates and infants. Therefore, when evaluating areas involving or adjacent to muscles, sedation to reduce motion before FDG injection and during the waiting period for PET may warrant more consideration. Figures 1, 2, 3 depict whole-body FDG uptake in neonates and infants, showing a lack of definitive artifacts related to patient movement during PET. However, in several cases, increased FDG uptake is observed in the muscles (neck, upper extremities and lower extremities), likely corresponding to uncontrolled movements around the time of FDG injection (Figs. 1, 2, 3). These movements may result in increased metabolic activity in the muscles and the uptake pattern is usually distinctive and symmetrical in various muscle groups.

The optimal injected dose of FDG is considered to be 3.7–5.2 MBq/kg per body for PET/CT (with a minimum of 26 MBq) and 3.7 MBq/kg for brain PET/CT (with a minimum of 14 MBq) as a scan protocol, and a guideline is available for CT doses [7]. The dose administered should be the lowest possible dose that will produce diagnostic-quality images. If a subject weighs 7 kg and is injected with 26–36 MBq of FDG, the effective dose can be estimated at less than 6.5 mSv [7].

The “SNMMI/EANM guideline for pediatric ^18^F-FDG PET/CT for Oncology” emphasizes the importance of emptying the bladder before FDG-PET to reduce bladder activity, which can result in increased radiation exposure and image artifacts. Bladder catheterization is not routinely recommended for PET/CT studies, but when tumors or lymph nodes are present near the urinary bladder and the patient is unable to voluntarily empty the bladder, catheterization or administration of intravenous furosemide can aid bladder emptying. In neonates and infants, ensuring the diaper is changed just before image acquisition is crucial to avoid artifacts and ensure the accuracy of PET. This practice minimizes any interference from urine, which can otherwise accumulate FDG and affect scan quality. Various shapes of FDG uptake in the bladder and FDG-containing urine in diapers were observed (Figs. 1, 2, 3).

**Fig. 4:**
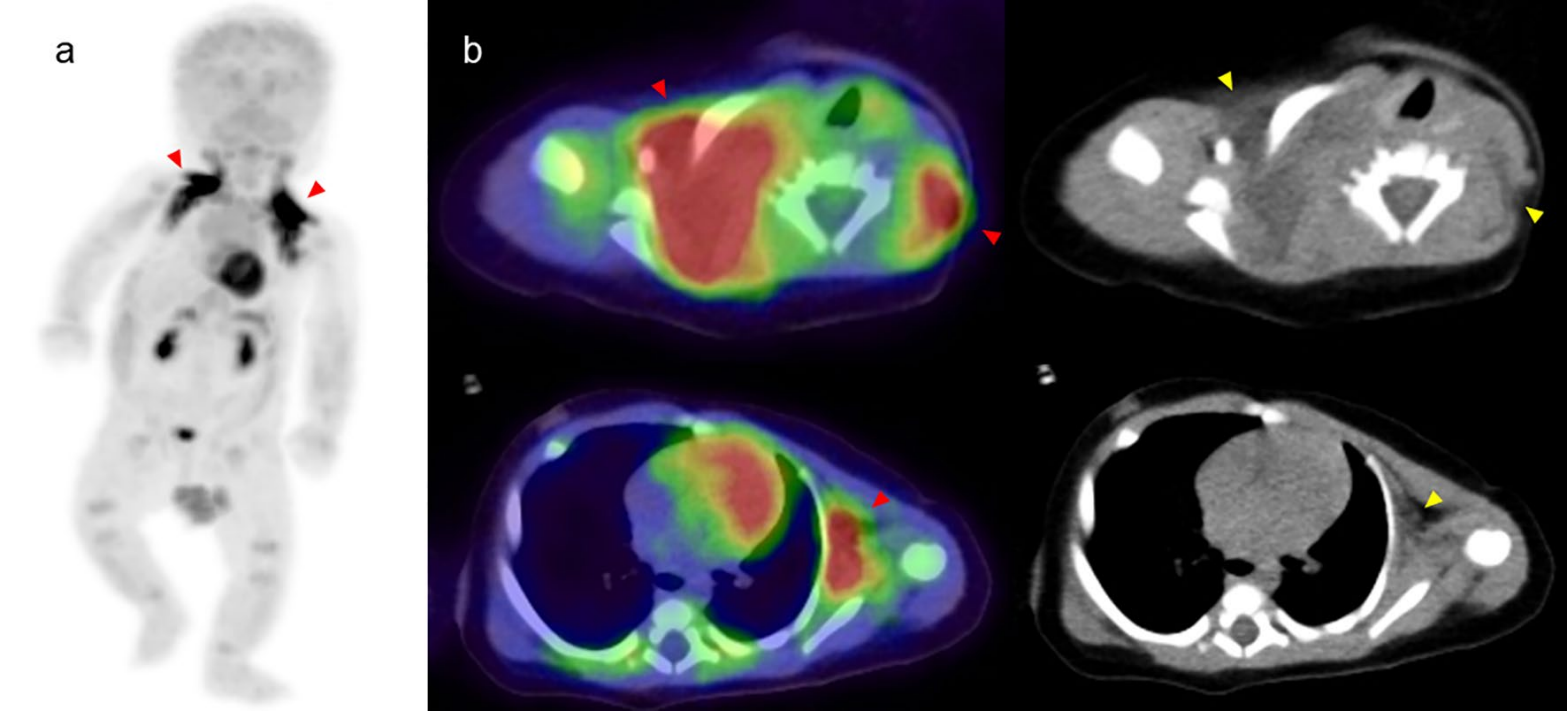
**a** Whole body FDG-PET image of infants with 51days after birth, **b** fused FDG-PET/CT, **c** CT portion of PET/CT. Significant FDG uptake is confirmed at bilateral neck, subclavian region and axillar region (red arrowhead) of those are corresponded to brown adipose tissue (yellow arrowhead)

Brown adipose tissue stores only small amounts of fat, primarily burning it to produce heat and help regulate body temperature. Neonates and infants have a much higher proportion of brown adipose tissue compared to adults, and this decreases gradually with age [14, 15]. FDG can accumulate in brown adipose tissue, normally showing bilateral, symmetrical uptake corresponding to fat tissue, and sometimes with focal and/or asymmetrical uptake (Fig. 4). The prevalence of detecting brown adipose tissue is significantly higher in pediatric PET/CT examinations (31–77%) compared to adults (5%) [15–18]. Neonates and infants should begin warming from half an hour prior to FDG injection and then continue during the uptake phase, ideally in a heated environment with blankets, to reduce radiotracer uptake into thermogenic brown adipose tissue that would impair the quality of imaging [18].

### Brain and spine

FDG uptake into the brain varies significantly with patient age. Brain glucose metabolism is diffusely lower in neonates and early infants compared to adults, with levels around 30% of those seen in adults [19, 20]. Brain glucose metabolism begins to increase rapidly after 4 months of age, peaking at levels approximately 30% higher than adult metabolism by around 4 years of age [21, 22].

In neonates and infants, particularly in the first 1–2 months of life, most of the cerebral cortex, except the primary sensorimotor cortex, and the basal ganglia show low metabolic activity. In preterm and term infants, local cerebral metabolic rates of glucose (LCMRGlc) in the primary sensorimotor cortex range from 4 to 16 μmol/100 g/min, while the frontal cortex shows lower activity (3.5–15 μmol/100 g/min), increasing with postconceptional age. LCMRGlc values in the temporal and occipital cortices are also low at birth (4–10 μmol/100 g/min) and correlate positively with advancing age [23]. The thalamus, brainstem, and cerebellar vermis exhibit the highest metabolic activity in neonates and infants under 2 months old [24, 25]. LCMRGlc is highest in the thalamus, brainstem, and sensorimotor cortex. Thalamic LCMRGlc values in the thalamus range from 5 to 20 μmol/100 g/min before 42 weeks of postconceptional age, rising to 35 μmol/100 g/min by 56–60 weeks. Similarly, cerebellar LCMRGlc ranges from 5–16 μmol/100 g/min in the first few months of life, increasing as the infant grows. The brainstem, which is already relatively advanced at birth, shows LCMRGlc values between 4 and 20 μmol/100 g/min, reaching 23 μmol/100 g/min by a postconceptional age of 56–60 weeks [23]. Whole-brain LCMRGlc correlates closely with both post-conceptional and postnatal age, with lower values observed in preterm babies compared to term babies at 50–60 postconceptional weeks [23]. Compared to preterm babies (mean gestational age, 33.5 ± 3.1 weeks), term babies (mean gestational age, 38.7 ± 1.6 weeks) shows higher FDG uptake in the thalamus (standardized uptake values: 0.71 ± 0.11 vs 1.74 ± 0.41), cerebellum (0.66 ± 0.13 vs 1.22 ± 0.21), sensorimotor cortex (0.55 ± 0.09 vs 1.09 ± 0.17), basal ganglia (0.51 ± 0.07 vs 1.01 ± 0.16), frontal region (0.44 ± 0.05 vs 0.93 ± 0.19), temporal region (0.44 ± 0.04 vs 0.73 ± 0.25) and occipital region (0.46 ± 0.05 vs 0.94 ± 0.18) [26].

**Fig. 5:**
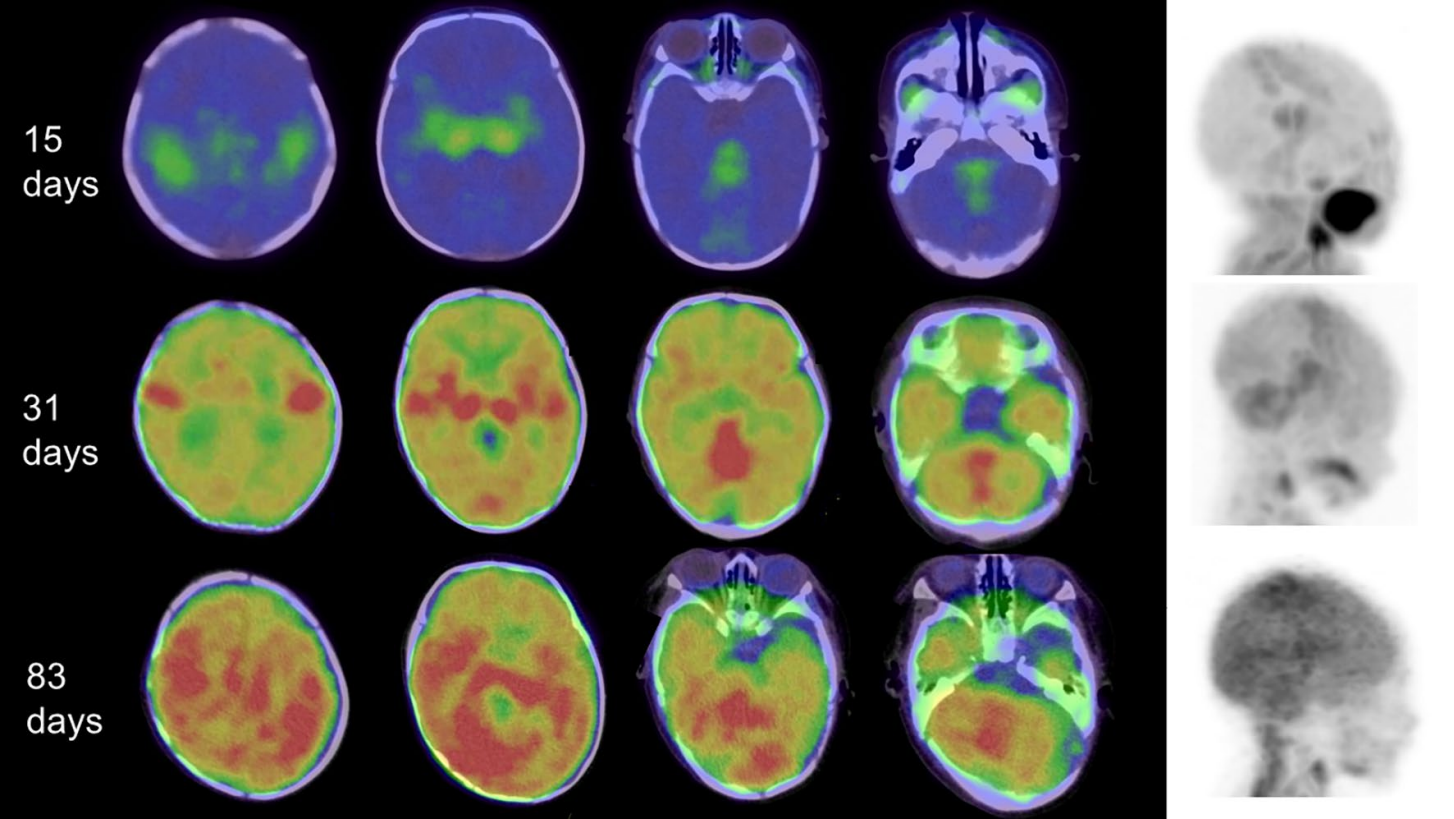
Alternation of brain FDG uptake from neonate to infant. In neonate (15 days after birth), FDG uptake for sensorimotor cortex (left end), thalamus (2^nd^ from the left), brain stem (3^rd^ from the left), cerebellar vermis (right end) are prominent. In infant with one month after the birth, slight increase in FDG uptake in the brain hemispheres. FDG uptake in the brain hemispheres becomes more prominent after 2 months from birth. In addition, CT imaging of neonates and infants reveals that the cranial bones are not yet fully fused, showing gaps at the sutures

In neonates, FDG uptake is predominantly concentrated in the sensorimotor cortex, thalamus, brainstem, cerebellar vermis, and cerebellar hemispheres, as shown in Figs. 1 and 5. Around 1 month after birth, the same pattern of FDG uptake is observed, but with a slight increase in FDG uptake in the brain hemispheres (Figs. 2 and 5). By 2 months after birth, FDG uptake in the brain hemispheres becomes more prominent (Figs. 3 and 5), becoming close to the levels of FDG uptake usually observed in the adult brain.

By around 3 months old, infants transition from random, uncoordinated movements to more coordinated ones. This developmental milestone is largely driven by improvements in visual-spatial and visual-motor integration, reflecting the activation of brain regions such as the frontal eye fields, parietal cortex, basal ganglia, and cerebellar cortex. At this stage, the ability of the infant to visually track objects and engage in hand-eye coordination becomes more refined, enabling more deliberate reaching for and interaction with objects. This shift reflects the maturation of several brain regions, including the parietal cortex, basal ganglia, and cerebellum, which are responsible for processing sensory input and coordinating motor responses [27]. By 5 months old, glucose metabolism has increased in the frontal, parietal, temporal, occipital, and cerebellar regions [21]. Between 6 and 8 months old, glucose metabolism increases in the frontal cortex beginning in the lateral and inferior portions and extending to the medial and superior portions by 1 year old [21]. At about 8 months old, an increase in CMRGlc becomes evident at the level of the dorsolateral and frontal occipital cortex, corresponding to enhanced interactions of the child with their surroundings in this period of life [27]. The characteristics of FDG uptake vary in cases of preterm infants, low birth weight, structural abnormality and abnormalities in glucose metabolism [20, 26–28]. CT imaging in infants shows an absence of continuous bone structure, with gaps visible at the cranial sutures and fontanelles.

**Fig. 6:**
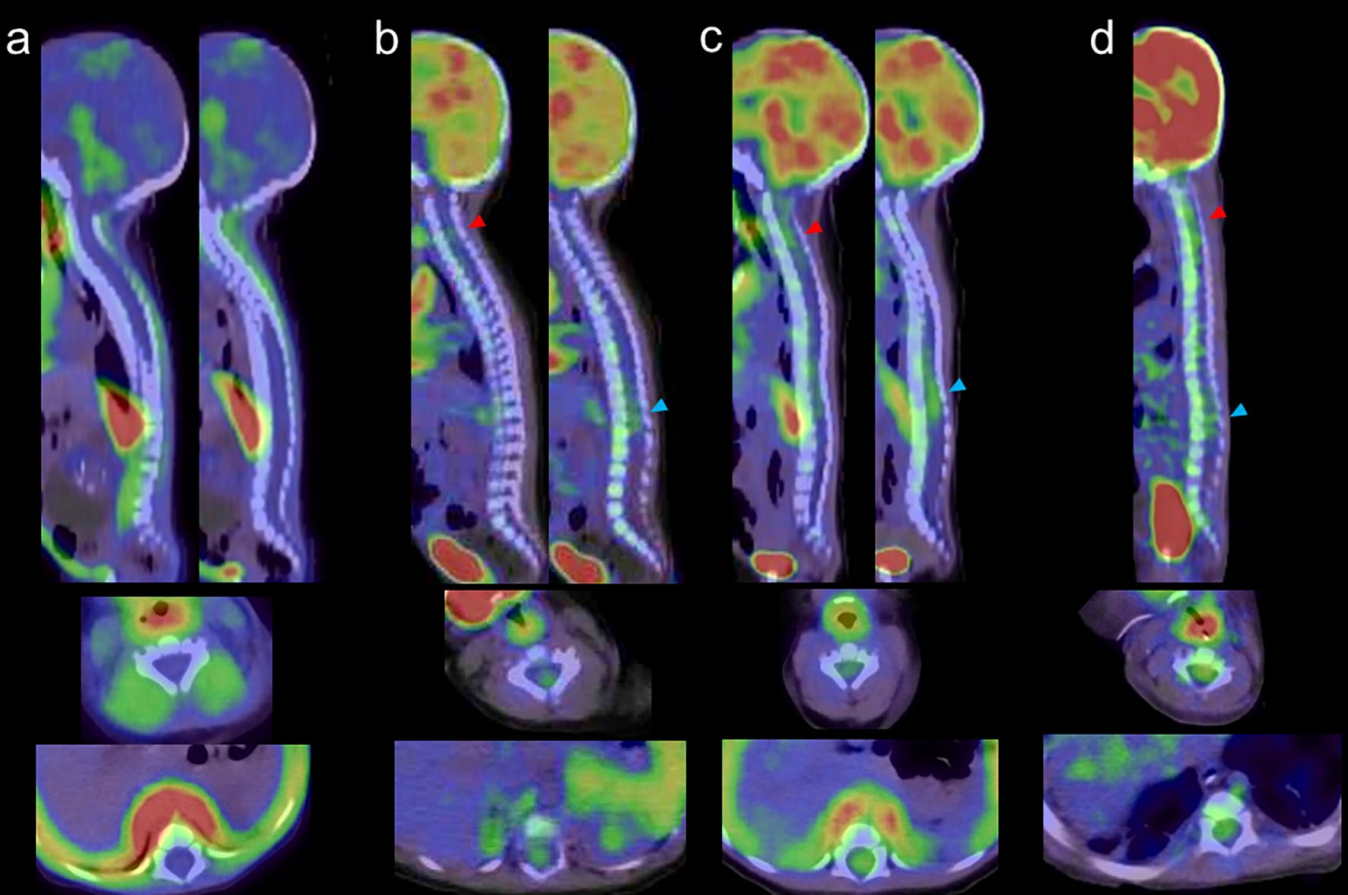
Sagittal and axial FDG-PET images in neonates and infants, **a** 15 days after birth, **b** 38 days after birth, **c** 61 days after birth, **d** 107 days after birth. FDG uptake in the cervical (red arrowhead) and lower thoracic spinal cord (blue arrowhead) is visible

Metabolic activity in the pons and spinal cord increases with age in children. Physiological FDG uptake in the spine is typically higher in the upper cervical region (level of the 4th cervical vertebra) and the lower thoracic region (level of the 11th and 12th thoracic vertebrae) compared to other areas [29]. However, physiological FDG uptake in spinal code is less common in children under 4.9 years old compared to those in children aged 5–18.9 years old [30]. We present FDG-PET/CT images of the spinal cord in neonates and infants (Fig. 6).

### Head and neck

**Fig. 7:**
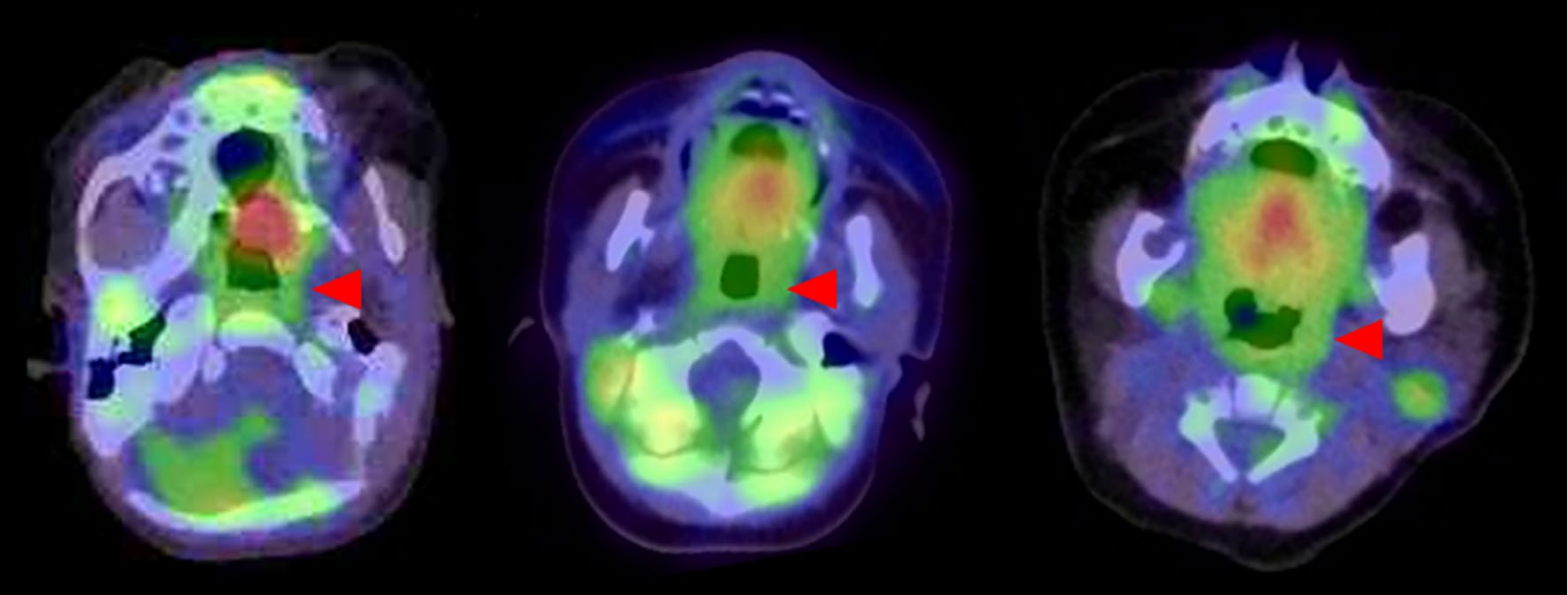
FDG PET/CT image of tonsil from neonates to infants. **a** 15 days after birth, **b** 38 days after birth, **c** 61 days after birth. Physiological intense FDG uptake in the and tonsils (red arrowhead) appears to be less frequent in the neonate and infants

In children without snoring, the adenoids and tonsils tend to grow proportionally to the surrounding skeletal structures, as observed in MRI studies, and do not typically restrict the pharyngeal airway [31]. Lateral skull radiographs show only a minimal adenoid shadow at 1 month old, becoming more pronounced by around 2 years old [32]. In non-snoring children, the growing adenotonsillar tissue narrows the upper airway to varying extents, but this narrowing mainly occurs during the first 8 years of life, after which the growth of the airway outpaces the expansion of adenotonsillar tissue, thus reducing the potential for impacts on airway obstruction [33]. Physiological FDG uptake in the adenoids and tonsils is therefore less frequent in the neonate (Fig. 7).

**Fig. 8:**
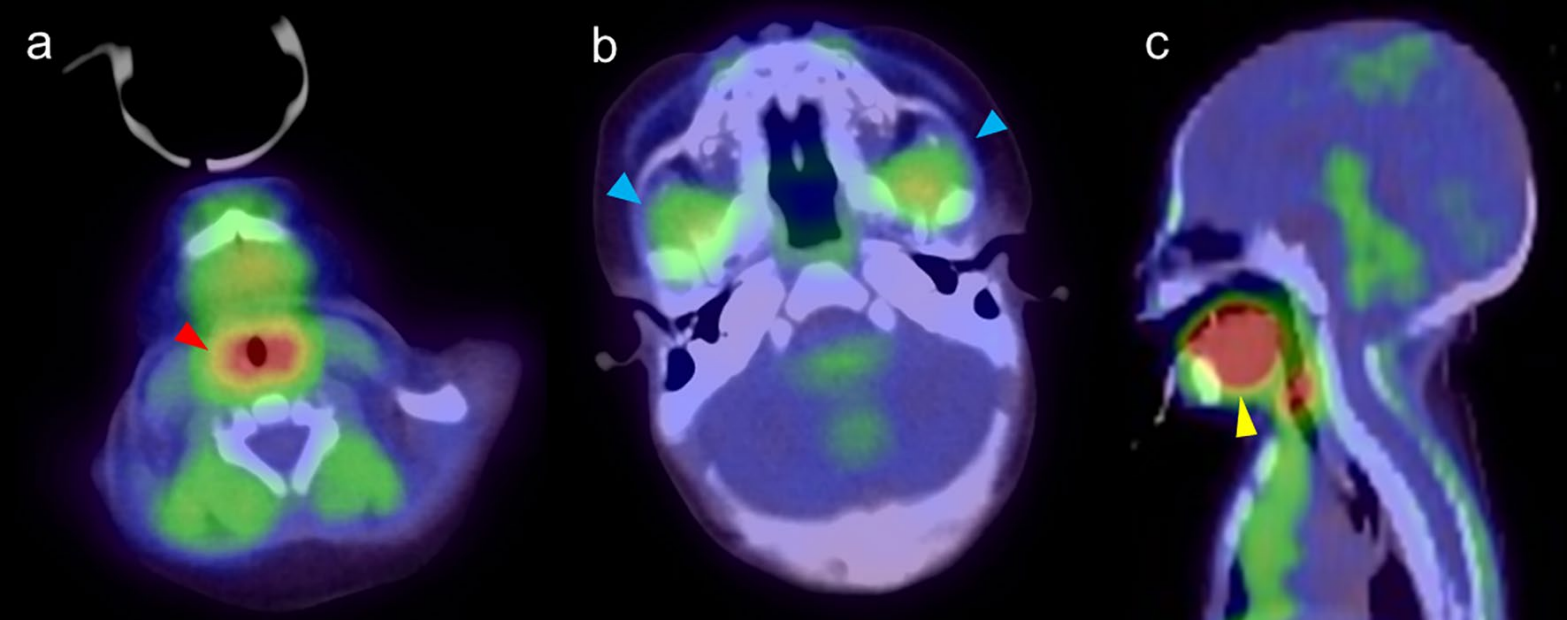
FDG PET/CT image of neonates with15 days after birth. **a** increased FDG uptake in the muscles associated with vocalization cause by crying (red arrowhead). **b** increased FDG uptake in masseter used for suck on pacifiers (blue arrowhead). **c** increased FDG uptake in tongue muscles used for suck on pacifier (yellow arrowhead)

FDG uptake in the laryngeal muscles can be observed if neonates or infants cry during the uptake phase of an FDG-PET scan [3]. This is due to the increased metabolic activity in the muscles associated with vocalization (Fig. 8a). When babies suck on pacifiers, the orbicularis oris, masseter, temporalis, buccinator, and lingual muscles work together to allow babies to suck effectively [34] (Fig. 8b and c). These muscles can show elevated FDG uptake in neonates and infants [2, 3, 35].

### Chest

In neonates, the ribs are elevated and positioned horizontally, resulting in little changes to intrathoracic volume during respiration, whereas adults show elevation of the ribs during inspiration to increase the intrathoracic volume [36]. Rib development starts around 9 weeks of gestation, with secondary ossification centers (SOCs) appearing around 15 years of age. By day 45, the first seven ribs are connected to the sternum via costal cartilages, while the lower five ribs remain unattached to the sternum [37]. The non-ossified and compliant nature of the neonatal chest wall makes the thorax more prone to deformation. The shape of the thoracic cage in the neonate is more conical compared to the cylindrical shape seen in adults [38].

**Fig. 9:**
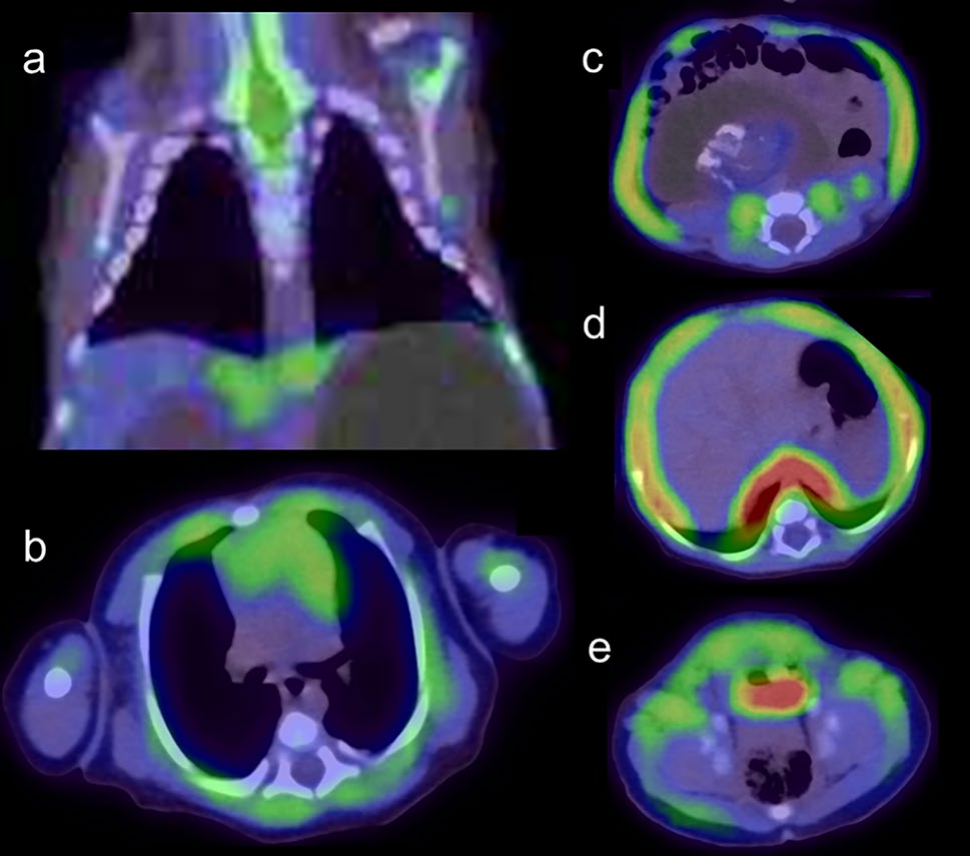
FDG PET/CT image of neonates with15 days after birth. **a** the neonatal diaphragm is flatter and attaches to the chest wall at a wider angle. **b** physiological FDG uptake in thymus, and intercostal muscles supporting breathing. **a**–**e** abdominal muscle supporting breathing and diaphragm

Unlike the dome shape seen in adults, the neonatal diaphragm is flatter and attaches to the chest wall at a wider angle, reducing the zone of apposition and limiting the range of displacement. This structural difference causes the diaphragm to function more like a bellows (Fig. 9a). In addition, the neonatal diaphragm has fewer slow-twitch, fatigue-resistant fibers, increasing the susceptibility to muscle fatigue. FDG-PET imaging in neonates can reveal the horizontal alignment of the ribs and a flatter diaphragm, representing characteristic morphologies. Uptake of FDG into the sternocleidomastoid, scalene, and abdominal muscles reflects the increased involvement of these muscles in neonatal breathing (Fig. 9b–e).

When breathing effort increases, accessory muscles such as the sternocleidomastoid and scalene muscles are recruited to prevent fatigue [36]. In healthy preterm neonates, abdominal muscles are also activated in response to CO_2_ rebreathing [39]. Moreover, mechanically ventilated infants or those experiencing respiratory distress show marked activity of the diaphragm and abdominal muscles during expiration [2, 40]. Uptake in the diaphragm, the crura of the diaphragm, and the intercostal muscles can be observed in neonates and infants who have been crying during FDG-PET (Fig. 9) [2].

### Thymus

The thymus plays a crucial role in T-cell development [41], and glucose metabolism is essential in supporting the energy demands of this process [42]. Thymocytes undergo several stages of differentiation, with varying metabolic needs during these stages. Early-stage thymocytes rely heavily on glycolysis for energy, then reduce their reliance on glycolysis and shift towards mitochondrial oxidative phosphorylation for energy production as they mature. This metabolic flexibility is necessary to support the survival, proliferation, and proper selection of functional T cells. Glucose transporter (Glut-1) expression on the cell surface is critical for maintaining high glucose uptake, supporting both glycolysis and mitochondrial oxidative phosphorylation [43]. The thymus begins to form early in gestation, with fetal T cells detectable as early as 8 weeks. The organ reaches its maximum size relative to total body weight shortly after birth, then begins to decrease in size [43, 44].

Diffuse and homogeneous uptake in the thymus is common in healthy children [2, 44]. In general, the thymus is visible on CT scans in almost all pediatric patients [45].

The exact timing of the initial appearance of FDG uptake in the thymus is not well established. However, our cases demonstrate that physiologic thymic uptake can be observed as early as the neonatal period (Fig. 9b). Generally, physiological uptake in the thymus disappears during adolescence in conjunction with the involution of the thymus. A study suggested that uptake typically disappears after puberty (around 13 years old) due to fatty infiltration and involution of the thymus [46]. However, later research confirmed that normal thymic uptake can persist beyond puberty, it can be also seen in a relatively large number of adult patients [47].

Intense thymic FDG uptake may also be seen following chemotherapy, reflecting thymic hyperplasia [48].

### Myocardium

**Fig. 10:**
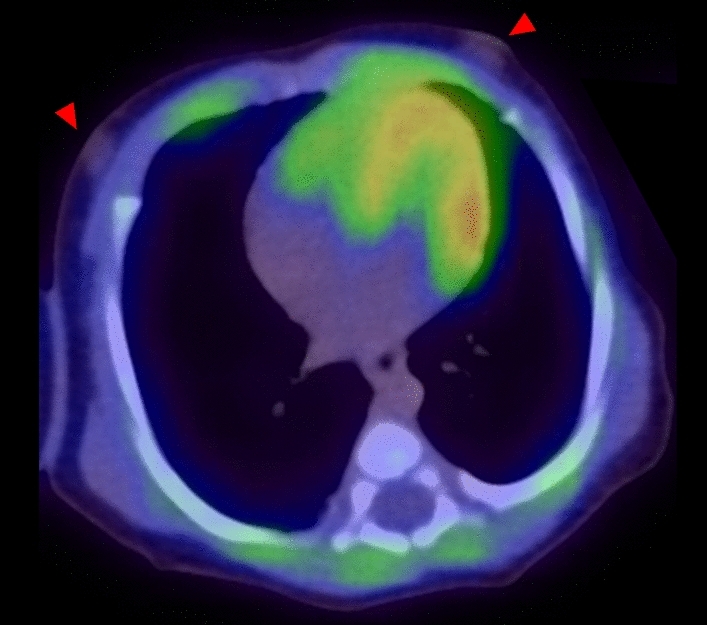
FDG PET/CT image of infants with 38 days after birth. Breast enlargement (red arrowhead) and FDG uptake in left ventricular wall

Lactate and glucose are important energy substrates for the heart. In the immediate neonate period, lactate oxidation and glycolysis are the preferred sources for ATP production, as noted in various reviews [49, 50]. The contribution of lactate and glucose to energy production is generally agreed to be higher in the fetal and immediate neonate hearts. The mechanisms for glucose and lactate transport differ significantly between the neonates and adult stages. During fetal life, glucose uptake is primarily regulated by the GLUT-1 transporter, but shortly after birth a shift occurs from the GLUT-1 isoform to the GLUT-4 isoform, the dominant glucose transporter in the adult heart [51]. However, fatty acid oxidation gradually increases after birth and becomes the predominant source of ATP production in the heart across most species [52]. The ability of the heart to oxidize fatty acids has been shown to increase within the first week of life in rabbits [53, 54]. Reflecting the metabolic changes in the myocardium during the early neonatal period, FDG uptake in the myocardium may fluctuate over a short period (Fig. 10).

### Breast

Neonatal breast enlargement refers to a benign, non-pathological proliferation of glandular tissue in neonates. This condition is believed to be caused by maternal estrogens crossing the placenta into the fetal circulation, or as a reaction to the sharp decline in maternal estrogen levels toward the end of pregnancy, which may trigger the pituitary gland in the neonate to secrete prolactin [55–57]. This phenomenon is observed in 60–90% of neonates and can be uni- or bilateral, with the bilateral presentation more common [57, 58]. The condition typically emerges during the first week of life and generally resolves spontaneously by the time the infant reaches 6 months of age, but may persist longer in some cases [56]. FDG-PET imaging studies have suggested that moderate and diffuse FDG uptake can occur in normal breast tissue due to the glandular proliferation [3]. However, we have not encountered any cases of FDG uptake related to neonatal breast enlargement (Fig. 10). FDG uptake in breast tissue tends to be more prevalent in older individuals and may not exhibit the same characteristics seen in neonates with spontaneous breast enlargement.

### Abdomen

FDG uptake by the liver appears to be lower in neonates and infants than in adults. FDG-PET uptake in the liver shows significant positive associations with age [59, 60] and body weight [60], but not with injected radiation dose or serum glucose level [59, 60]. Metabolic changes occur postnatally as the infant transitions from receiving glucose via the placenta to generating glucose through hepatic glycogenolysis and gluconeogenesis. After birth, blood glucose levels decrease in the neonate and stabilization of these levels depends on the activation of hepatic glycogenolysis and gluconeogenesis in response to changes in hormones such as insulin, cortisol, glucagon, and catecholamines [61]. In the early days of life, glucose production stabilizes as enteral feeding begins and hepatic gluconeogenesis continues to mature [61, 62]. Gluconeogenesis in the neonate liver can contribute to 30–70% of the body’s glucose production shortly after birth [63, 64].

The expression of GLUT2, a key glucose transporter, undergoes significant changes during the perinatal period. In fetal development, GLUT1 is the predominant glucose transporter in hepatocytes, facilitating glucose uptake necessary for growth and energy storage. After birth, there is a marked decrease in GLUT1 expression and a corresponding increase in GLUT2 levels in the liver. GLUT2 is the primary glucose transporter in the adult liver; therefore, the FDG uptake level in the liver of infants and neonates may be influenced by the developmental transition of GLUT2 expression [65, 66].

FDG uptake in the intestine and colon seems to be low in neonates and infants. The maturated gastrointestinal system in full-term infants is able to acquire adequate amounts of nutrients to promote the rapid growth that occurs shortly after birth [67]. Currently, it remains unclear whether the observed low FDG uptake in the intestine and colon are specific to neonates and infants, who generally digest and absorb nutrients from either breast milk or infant formula. No other distinctive patterns of FDG uptake have been identified in the abdominal region in our experience and from a search of the literature. A more detailed investigation is required to draw conclusive findings on the abdominal area.

## Growth plates and secondary ossification centers

**Fig. 11:**
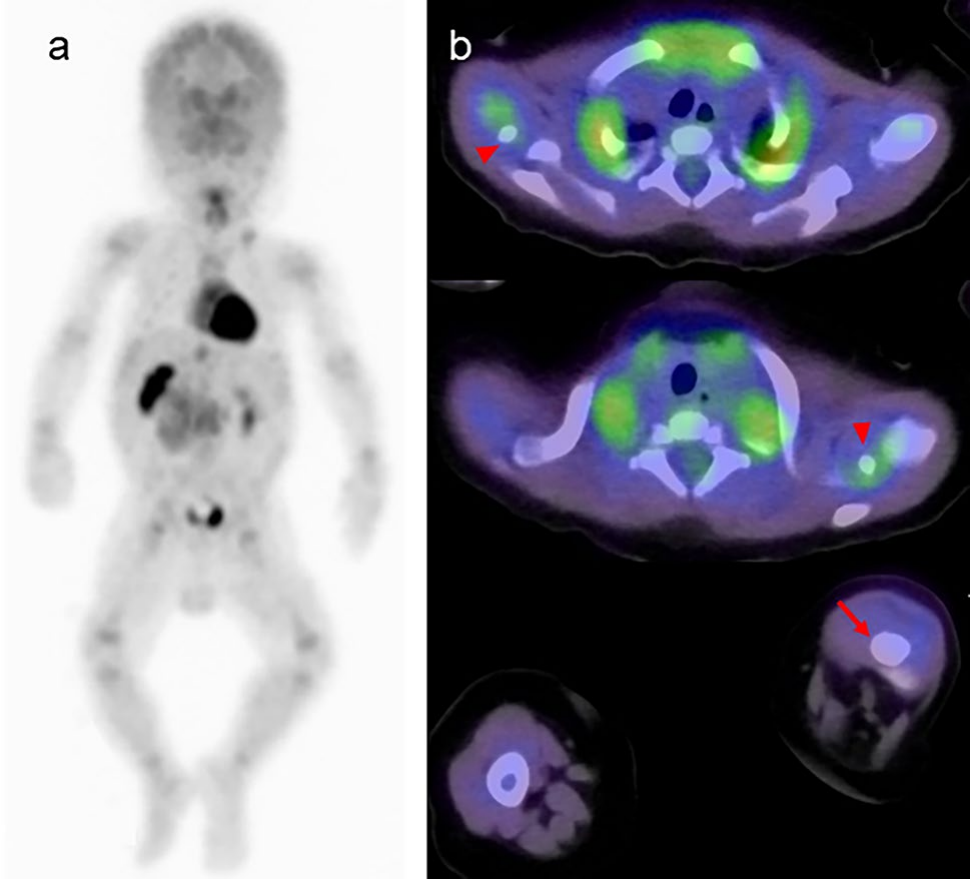
FDG PET/CT image of infants with 31 days after birth. **a** horizontal band like FDG uptake in the growth plates. **b** Secondary ossification centers (SOCs) shows slight FDG uptake at the femoral head (arrow head) and no specific FDG uptake at the proximal left tibia (arrow)

The growth plates, located at each end of the long bones, are regions where new bone formation occurs. SOCs in the distal femur and proximal tibia develop during the perinatal period, while those in other long bones typically appear after birth. From these centers, bone growth spreads outward through a process called centrifugal growth, leading to rapid ossification from the growth plate [68]. As children mature, the growth plates gradually ossify and close, leading to the end of bone growth. Physiological FDG uptake is commonly seen in the growth plates of pediatric patients and varies according to age and the specific location of the growth plate. The highest uptake is typically observed in the distal femur, appearing as a horizontal band of increased FDG uptake in the physes and apophyses (Fig. 11). This uptake is generally bilateral and symmetrical, reflecting active bone growth in these regions [69].

The age of appearance and fusion of the SOCs varies in each bone. SOCs appear early in the humeral head (0.5–2 years old), femoral head (0.5–1 year old), distal end of the femur (36–40 weeks old), proximal tibia (36 weeks–2 months old), and distal tibia (3–10 months old) [70]. In our observations, we identified SOC on CT, with or without slight FDG uptake, in the femoral head and at the distal end of the femur or proximal tibia (Fig. 11).

Focal FDG uptake may be observed in the upper or lower extremities on FDG-PET/CT images as a result of recent immunizations [71, 72].

## Conclusion

In this review, we examined the unique characteristics of FDG-PET imaging in neonates and infants. This age group is in a critical phase of rapid growth and development, exhibiting significant physiological and metabolic differences compared to older children and adults. These distinctions impact FDG uptake patterns and the interpretation of PET/CT images, requiring tailored imaging protocols and careful consideration of normal developmental variations.

## References

[1] Güemes M, Rahman SA, Hussain K. What is a normal blood glucose? Arch Dis Child. 2016;101:569–74. 26369574 10.1136/archdischild-2015-308336

[2] Shammas A, Lim R, Charron M. Pediatric FDG PET/CT: physiologic uptake, normal variants, and benign conditions. Radiographics. 2009;2:1467–86. 10.1148/rg.295085247 19755606

[3] Stauss J, Franzius C, Pfluger T, Juergens KU, Biassoni L, Begent J, et al. Guidelines for ^18^F-FDG PET and PET-CT imaging in paediatric oncology. Eur J Nucl Med Mol Imaging. 2008;35:1581–8. 18536914 10.1007/s00259-008-0826-x

[4] Barrington SF, Begent J, Lynch T, Schleyer P, Biassoni L, Ramsden W, et al. Guidelines for the use of PET-CT in children. Nucl Med Commun. 2008;29:418–24. 18391724 10.1097/MNM.0b013e3282f767b2

[5] van der Walt JH, Foate JA, Murrell D, Jacob R, Bentley M. A study of preoperative fasting in infants aged less than three months. Anaesth Intensive Care. 1990;18:527–31. 2268020 10.1177/0310057X9001800420

[6] Zhang E, Hauser N, Sommerfield A, Sommerfield D, von Ungern-Sternberg BS. A review of pediatric fasting guidelines and strategies to help children manage preoperative fasting. Paediatr Anaesth. 2023;33:1012–9. 37533337 10.1111/pan.14738 PMC10947285

[7] Vali R, Alessio A, Balza R, Borgwardt L, Bar-Sever Z, Czachowski M, et al. SNMMI Procedure Standard/EANM Practice Guideline on Pediatric 18F-FDG PET/CT for Oncology 1.0. J Nucl Med. 2021;6:99–110. 10.2967/jnumed.120.254110 PMC8679588 33334912

[8] Camoni L, Santos A, Luporsi M, Grilo A, Pietrzak A, Gear J, et al. EANM procedural recommendations for managing the paediatric patient in diagnostic nuclear medicine. Eur J Nucl Med Mol Imaging. 2023;50:3862–79. 37555902 10.1007/s00259-023-06357-3 PMC10611649

[9] Juengling FD, Kassubek J, Martens-Le Bouar H, Reinhardt MJ, Krause T, Nitzsche EU, et al. Cerebral regional hypometabolism caused by propofol-induced sedation in children with severe myoclonic epilepsy: a study using fluorodeoxyglucose positron emission tomography and statistical parametric mapping. Neurosci Lett. 2002;335:79–82. 12459503 10.1016/s0304-3940(02)01060-1

[10] Mandell G MM, Shalaby-Rana E, Gordon I. Society of Nuclear Medicine procedure guideline for pediatric sedation in nuclear medicine. amazonaws website. http://s3.amazonaws.com/rdcms-snmmi/files/production/public/pg_ch31_0703. pdf. 2003;3:173–5. 9379206

[11] Kai CM, Ingvardsen B, Lemvig P, Sehested LT, Søndergaard LR, Møller S, et al. Successful paediatric renography does not require sedation. Dan Med J. 2019;66:A5542. 31066354

[12] Lasnon C, Coudrais N, Houdu B, Nganoa C, Salomon T, Enilorac B, et al. How fast can we scan patients with modern (digital) PET/CT systems? Eur J Radiol. 2020;129:109144. 32593080 10.1016/j.ejrad.2020.109144

[13] Zhou X, Xue S, Li L, Seifert R, Dong S, Chen R, Huang G, et al. Sedation-free pediatric [^18^F]FDG imaging on totalbody PET/CT with the assistance of artificial intelligence. Eur J Nucl Med Mol Imaging. 2024;51:4062–72. 38958680 10.1007/s00259-024-06818-3

[14] Carter BW, Schucany WG. Brown adipose tissue in a newborn. Proc (Bayl Univ Med Cent). 2008;21:328–30. 18628932 10.1080/08998280.2008.11928419 PMC2446425

[15] Gilsanz V, Hu HH, Kajimura S. Relevance of brown adipose tissue in infancy and adolescence. Pediatr Res. 2013;73:3–9. 23090604 10.1038/pr.2012.141 PMC3614088

[16] Gelfand MJ, O’Hara SM, Curtwright LA, Maclean JR. Pre-medication to block [^18^F]FDG uptake in the brown adipose tissue of pediatric and adolescent patients. Pediatr Radiol. 2005;35:984–90. 15988582 10.1007/s00247-005-1505-8

[17] Drubach LA, Palmer EL 3rd, Connolly LP, Baker A, Zurakowski D, Cypess AM. Pediatric brown adipose tissue: detection, epidemiology, and differences from adults. J Pediatr. 2011;159:939–44. 21839465 10.1016/j.jpeds.2011.06.028

[18] Zukotynski KA, Fahey FH, Laffin S, Davis R, Treves ST, Grant FD, et al. Constant ambient temperature of 24 degrees C significantly reduces FDG uptake by brown adipose tissue in children scanned during the winter. Eur J Nucl Med Mol Imaging. 2009;36:602–6. 19037639 10.1007/s00259-008-0983-y

[19] Chugani HT. Imaging Brain Metabolism in the Newborn. J Child Neurol. 2018;33:851–60. 30112963 10.1177/0883073818792308

[20] Stanescu L, Ishak GE, Khanna PC, Biyyam DR, Shaw DW, Parisi MT. FDG PET of the brain in pediatric patients: imaging spectrum with MR imaging correlation. Radiographics. 2013;33:1279–303. 24025925 10.1148/rg.335125152

[21] Chugani HT, Phelps ME. Imaging human brain development with positron emission tomography. J Nucl Med. 1991;32:23–6. 1988631

[22] Kennedy C, Sokoloff L. An adaptation of the nitrous oxide method to the study of the cerebral circulation in children: normal values for cerebral blood flow and cerebral metabolic rate in childhood. J Clin Invest. 1957;36:1130–7. 13449166 10.1172/JCI103509 PMC1072700

[23] Kinnala A, Suhonen-Polvi H, Aärimaa T, Kero P, Korvenranta H, Ruotsalainen U, et al. Cerebral metabolic rate for glucose during the first six months of life: an FDG positron emission tomography study. Arch Dis Child Fetal Neonatal Ed. 1996;74:F153-7. 8777676 10.1136/fn.74.3.f153 PMC2528340

[24] Chugani HT, Phelps ME, Mazziotta JC. Positron emission tomography study of human brain functional development. Ann Neurol. 1987;22:487–97. 3501693 10.1002/ana.410220408

[25] Chugani HT. Metabolic imaging: a window on brain development and plasticity. Neuroscientist. 1999;5:29–40.

[26] Shi Y, Jin RB, Zhao JN, Tang SF, Li HQ, Li TY. Brain positron emission tomography in preterm and term newborn infants. Early Hum Dev. 2009;85:429–32. 19269116 10.1016/j.earlhumdev.2009.02.002

[27] Cacciatore M, Grasso EA, Tripodi R, Chiarelli F. Impact of glucose metabolism on the developing brain. Front Endocrinol. 2022;13:1047545. 10.3389/fendo.2022.1047545 PMC9816389 36619556

[28] Park JH, Kim CS, Won KS, Oh JS, Kim JS, Kim HW. Asymmetry of cerebral glucose metabolism in very low-birth-weight infants without structural abnormalities. PLoS ONE. 2017;12:e0186976. 29095842 10.1371/journal.pone.0186976 PMC5667759

[29] McCarville MB, Monu N, Smeltzer MP, Li CS, Laningham FH, Morris EB, et al. PET-CT of the normal spinal cord in children. Acad Radiol. 2009;16:881–5. 19427802 10.1016/j.acra.2009.01.022 PMC3680129

[30] Taralli S, Leccisotti L, Mattoli MV, Castaldi P, de Waure C, Mancuso A, et al. Physiological Activity of Spinal Cord in Children: An 18F-FDG PET-CT Study. Spine. 2015;40:E647-52. 25803218 10.1097/BRS.0000000000000895

[31] Arens R, McDonough JM, Corbin AM, Hernandez ME, Maislin G, Schwab RJ, et al. Linear dimensions of the upper airway structure during development: assessment by magnetic resonance imaging. Am J Respir Crit Care Med. 2002;165:117–22. 11779740 10.1164/ajrccm.165.1.2107140

[32] Capitanio MA, Kirkpatrick JA. Nasopharyngeal lymphoid tissue: roentgen observations in 257 children two years of age or less. Radiology. 1970;96:389–91. 5431426 10.1148/96.2.389

[33] Papaioannou G, Kambas I, Tsaoussoglou M, Panaghiotopoulou-Gartagani P, Chrousos G, Kaditis AG. Age-dependent changes in the size of adenotonsillar tissue in childhood: implications for sleep-disordered breathing. J Pediatr. 2013;162:269-74.e4. 22939928 10.1016/j.jpeds.2012.07.041

[34] Eishima K. The analysis of sucking behaviour in newborn infants. Early Hum Dev. 1991;27:163–73. 1802669 10.1016/0378-3782(91)90192-6

[35] Tong C, Zhuang H. Increased Genioglossus Muscle FDG Activity Due to Using Pacifier. Clin Nucl Med. 2022;47:655–7. 35195586 10.1097/RLU.0000000000004105

[36] Dassios T, Vervenioti A, Dimitriou G. Respiratory muscle function in the newborn: a narrative review. Pediatr Res. 2022;91:795–803. 33875805 10.1038/s41390-021-01529-z PMC8053897

[37] Glass RB, Norton KI, Mitre SA, Kang E. Pediatric ribs: a spectrum of abnormalities. Radiographics. 2002;22:87–104. 11796901 10.1148/radiographics.22.1.g02ja1287

[38] Vilensky JA, Suárez-Quian CA. Newborn anatomy. Clin Anat. 2022;35:15–8. 34378242 10.1002/ca.23774

[39] Praud JP, Egreteau L, Benlabed M, Curzi-Dascalova L, Nedelcoux H, Gaultier C. Abdominal muscle activity during CO2 rebreathing in sleeping neonates. J Appl Physiol. 1985;1991:1344–50. 10.1152/jappl.1991.70.3.1344 2033002

[40] South M, Morley CJ, Hughes G. Expiratory muscle activity in preterm babies. Arch Dis Child. 1987;62:825–9. 3310917 10.1136/adc.62.8.825 PMC1778477

[41] Miller JF. The golden anniversary of the thymus. Nat Rev Immunol. 2011;11:489–95. 21617694 10.1038/nri2993

[42] Elhage R, Kelly M, Goudin N, Megret J, Legrand A, Nemazanyy I, et al. Mitochondrial dynamics and metabolic regulation control T cell fate in the thymus. Front Immunol. 2024;14:1270268. 38288115 10.3389/fimmu.2023.1270268 PMC10822881

[43] Aaby P, Marx C, Trautner S, Rudaa D, Hasselbalch H, Jensen H, et al. Thymus size at birth is associated with infant mortality: a community study from Guinea-Bissau. Acta Paediatr. 2002;91:698–703. 12162605 10.1080/080352502760069142

[44] Moore SE, Fulford AJ, Wagatsuma Y, Persson LÅ, Arifeen SE, Prentice AM. Thymus development and infant and child mortality in rural Bangladesh. Int J Epidemiol. 2014;43:216–23. 24366492 10.1093/ije/dyt232 PMC3937977

[45] Ferdinand B, Gupta P, Kramer EL. Spectrum of thymic uptake at ^18^F-FDG PET. Radiographics. 2004;24:1611–6. 15537971 10.1148/rg.246045701

[46] Patel PM, Alibazoglu H, Ali A, Fordham E, LaMonica G. Normal thymic uptake of FDG on PET imaging. Clin Nucl Med. 1996;21:772–5. 8896924 10.1097/00003072-199610000-00004

[47] Jerushalmi J, Frenkel A, Bar-Shalom R, Khoury J, Israel O. Physiologic thymic uptake of ^18^F-FDG in children and young adults: a PET/CT evaluation of incidence, patterns, and relationship to treatment. J Nucl Med. 2009;50:849–53. 19443604 10.2967/jnumed.108.058586

[48] Brink I, Reinhardt MJ, Hoegerle S, Altehoefer C, Moser E, Nitzsche EU. Increased metabolic activity in the thymus studied with FDG PET: age dependency and frequency after chemotherapy. J Nucl Med. 2001;42:591–5. 11337547

[49] Lopaschuk GD, Collins-Nakai RL, Itoi T. Developmental changes in energy substrate use by the heart. Cardiovasc Res. 1992;26:1172–80. 1288863 10.1093/cvr/26.12.1172

[50] Makinde AO, Kantor PF, Lopaschuk GD. Maturation of fatty acid and carbohydrate metabolism in the newborn heart. Mol Cell Biochem. 1998;188:49–56. 9823010

[51] Postic C, Leturque A, Prinz RL, Maulard P, Loizeau M, Granner DK, et al. Development and regulation of glucose transporter and hexokinase expression in rat. Am J Physiol. 1994;266:E548-59. 8178975 10.1152/ajpendo.1994.266.4.E548

[52] Onay-Besikci A. Regulation of cardiac energy metabolism in newborn. Mol Cell Biochem. 2006;287:1–11. 16670818 10.1007/s11010-006-9123-9

[53] Lopaschuk GD, Spafford MA. Energy substrate utilization by isolated working hearts from newborn rabbits. AmJ Physiol. 1990;258:H1274-80. 2337162 10.1152/ajpheart.1990.258.5.H1274

[54] Lopaschuk GD, Spafford MA, Marsh DR. Glycolysis is predominant source of myocardial ATP production immediately after birth. Am J Physiol. 1991;261:H1698–705. 1750528 10.1152/ajpheart.1991.261.6.H1698

[55] Amer A, Fischer H. Images in clinical medicine Neonatal breast enlargement. N Engl J Med. 2009;360:1445. 19339724 10.1056/NEJMicm0707832

[56] D’Auria D, Ferrara D, Aragione N, De Chiara C, Argenziano G, Noschese I, et al. Role of ultrasound in diagnosis of neonatal breast enlargement: a newborn case report. Radiol Case Rep. 2021;16:2692–6. 34336074 10.1016/j.radcr.2021.06.057 PMC8318999

[57] Raveenthiran V. Neonatal mastauxe (breast enlargement of the newborn). J Neonatal Surg. 2013;2:31. 26023451 PMC4422278

[58] Johnson RE, Murad MH. Gynecomastia: Pathophysiology, evaluation, and management. Mayo Clin Proc. 2009;84:1010–5. 19880691 10.1016/S0025-6196(11)60671-X PMC2770912

[59] Cao Y, Zhou K, Diao W, Long X, Tian F, Su M, et al. Age-related changes of standardized uptake values in the blood pool and liver: a decade-long retrospective study of the outcomes of 2,526 subjects. Quant Imaging Med Surg. 2021;11:95–106. 33392014 10.21037/qims-20-35 PMC7719952

[60] Debnath P, Trout AT. Patient factors affecting ^18^F FDG uptake in children. Clin Imaging. 2024;107:110093. 38295511 10.1016/j.clinimag.2024.110093

[61] Hume R, Burchell A, Williams FL, Koh DK. Glucose homeostasis in the newborn. Early Hum Dev. 2005;81:95–101. 15707720 10.1016/j.earlhumdev.2004.10.005

[62] Hoseth E, Joergensen A, Ebbesen F, Moeller M. Blood glucose levels in a population of healthy, breast fed, term infants of appropriate size for gestational age. Arch Dis Child Fetal Neonatal Ed. 2000;83:F117-9. 10952705 10.1136/fn.83.2.F117 PMC1721132

[63] Girard J. Gluconeogenesis in late fetal and early neonatal life. Biol Neonate. 1986;50:237–58. 3542066 10.1159/000242605

[64] Kalhan S, Parimi P. Gluconeogenesis in the fetus and neonate. Semin Perinatol. 2000;24:94–106. 10805165 10.1053/sp.2000.6360

[65] Lane RH, Crawford SE, Flozak AS, Simmons RA. Localization and quantification of glucose transporters in liver of growth-retarded fetal and neonatal rats. Am J Physiol. 1999;276:E135-42. 9886959 10.1152/ajpendo.1999.276.1.E135

[66] Sadiq HF, deMello DE, Devaskar SU. The effect of intrauterine growth restriction upon fetal and postnatal hepatic glucose transporter and glucokinase proteins. Pediatr Res. 1998;43:91–100. 9432118 10.1203/00006450-199801000-00014

[67] Indrio F, Neu J, Pettoello-Mantovani M, Marchese F, Martini S, Salatto A, et al. Development of the Gastrointestinal Tract in Neonates as a Challenge for an Appropriate Nutrition: A Narrative Review. Nutrients. 2022;28:1405. 10.3390/nu14071405 PMC9002905 35406018

[68] Obgyn Key Growth Plate anatomy. https://obgynkey.com/growth-plate-anatomy/

[69] Purbhoo K, Vangu MD-T. Normal Variants and Pitfalls of ^18^F-FDG PET/CT Imaging in Pediatric Oncology. Front Nucl Med. 2022;2:825891. 39354970 10.3389/fnume.2022.825891 PMC11440973

[70] Augusto ACL, Goes PCK, Flores DV, Costa MAF, Takahashi MS, Rodrigues ACO, et al. Imaging Review of Normal and Abnormal Skeletal Maturation. Radiographics. 2022;42:861–79. 35213260 10.1148/rg.210088

[71] Sheehy N, Drubach L. ^18^F-FDG uptake at vaccination site. Pediatr Radiol. 2008;38:246. 18075737 10.1007/s00247-007-0686-8

[72] Galloway TL, Johnston MJ, Starsiak MD, Silverman ED. A Unique Case of Increased ^18^F-FDG Metabolic Activity in the Soft Tissues of the Bilateral Upper Thighs Due to Immunizations in a Pediatric Patient. World J Nucl Med. 2017;16:59–61. 28217022 10.4103/1450-1147.176886 PMC5314666

